# Artificial Intelligence in Surgery: A Systematic Review of Use and Validation

**DOI:** 10.3390/jcm13237108

**Published:** 2024-11-24

**Authors:** Nitzan Kenig, Javier Monton Echeverria, Aina Muntaner Vives

**Affiliations:** 1 Department of Plastic Surgery, Quironsalud Palmaplanas Hospital, 07010 Palma, Spain; 2 Department of Plastic Surgery, Albacete University Hospital, 02006 Albacete, Spain; 3 Department Otolaryngology, Son Llatzer University Hospital, 07198 Palma, Spain

**Keywords:** artificial intelligence, machine learning, surgery, validation, human-machine interaction

## Abstract

**Background**: Artificial Intelligence (AI) holds promise for transforming healthcare, with AI models gaining increasing clinical use in surgery. However, new AI models are developed without established standards for their validation and use. Before AI can be widely adopted, it is crucial to ensure these models are both accurate and safe for patients. Without proper validation, there is a risk of integrating AI models into practice without sufficient evidence of their safety and accuracy, potentially leading to suboptimal patient outcomes. In this work, we review the current use and validation methods of AI models in clinical surgical settings and propose a novel classification system. **Methods**: A systematic review was conducted in PubMed and Cochrane using the keywords “validation”, “artificial intelligence”, and “surgery”, following PRISMA guidelines. **Results**: The search yielded a total of 7627 articles, of which 102 were included for data extraction, encompassing 2,837,211 patients. A validation classification system named Surgical Validation Score (SURVAS) was developed. The primary applications of models were risk assessment and decision-making in the preoperative setting. Validation methods were ranked as high evidence in only 45% of studies, and only 14% of the studies provided publicly available datasets. **Conclusions**: AI has significant applications in surgery, but validation quality remains suboptimal, and public data availability is limited. Current AI applications are mainly focused on preoperative risk assessment and are suggested to improve decision-making. Classification systems such as SURVAS can help clinicians confirm the degree of validity of AI models before their application in practice.

## 1. Introduction

### 1.1. Description of the Conditions

Artificial Intelligence (AI) holds the prospect of advancing medicine in the coming years [1]. While AI algorithms have the potential to significantly enhance surgical decision-making, peri-operative evaluation, patient education, and communication, there is scarce consensus on how to adequately validate and use this new technology [2,3]. Despite their capabilities, AI models require rigorous validation to ensure their reliability and effectiveness in real-world applications, but they do not fall under regulations for surgical instruments or medication and can therefore often be applied under physicians’ criteria before official approval. Currently, the critical issue of validation can be undervalued or overlooked, while there is growing discussion about the significance and need for proper validation guidelines [4,5,6,7,8]. Guideline initiatives such as the European Guidelines on Minimally Invasive Pancreatic Surgery (EGUMIPS) have been suggested, but they apply only to a specific area of surgery and offer limited information for AI models [9]. Other concerns with AI applications refer to the generalizability of models, the scarcity of external validation [10,11,12], and the need for ethical guidelines [13]. Guidelines for AI such as CONSORT-AI and SPIRIT-AI [14] have been set forth, but they refer to research and publication guidelines, not to validation or use in the clinical setting.

AI models are being integrated into surgery, but quality research is a current concern [15]. The range of applications of AI has become increasingly widespread, including areas such as pre-operative complication prediction, but the need for validation has become pressing [16]. Image interpretation is another expanding field, but model performance in this area is also variable, with most studies lacking external validation [17]. Enthusiasm is present in different areas, including anatomy recognition [18], surgical decision making [19], and analysis of surgical videos [20], but clinical certification is an often unmet prerequisite [21]. The need for greater scientific rigor prior to deploying AI models and concerns about replication of results are other rising issues [22,23].

Due to the recent nature of the rise of AI models, comprehensive reviews of their current use and validation are lacking. In this work, we aim to outline their uses and applications in surgery and offer an extensive review of current validation methods. We propose a novel validation quality score for AI models in surgery, named SURVAS (Surgery Validation Score). SURVAS was developed as a classification system that is meant to help clinicians understand the degree to which AI models are validated for use in surgery.

### 1.2. Description of the Intervention

AI models are sets of algorithms, typically based on artificial neural networks, that use complex mathematical calculations to perform predictive tasks based on prior training with large datasets, enabling them to predict outputs for each given input. These models can perform tasks that previously required human intervention, such as decision-making and outcome prediction based on given data. In healthcare, particularly in surgery, AI models contribute to the clinical process across different stages by predicting surgical outcomes, assessing risks, and evaluating postoperative results, among various other tasks. For this study, AI model intervention is defined as any task involving data analysis through AI or machine-learning algorithms that generate predictions based on the analyzed data.

### 1.3. Why It Is Important to Conduct This Review

AI technology is poised to rapidly transform healthcare, offering significant advantages for improving patient care and efficiency. However, there is currently limited knowledge regarding the use and validation systems for AI models. In the literature, reviews of AI model applications in surgery are both limited and inconsistent, and there is a lack of standardized validation systems. This emphasizes the need to evaluate the reported uses and validation methods of AI models in surgical practice.

### 1.4. Objectives

The objective of this work is to review the current uses and validation methods of AI models in clinical surgery and to propose a novel classification system for validation methods.

## 2. Materials and Methods

This systematic review with a meta-analysis was conducted according to the PRISMA guidelines [24] and has been registered in PROSPERO with the following ID: CRD42024603176.

### 2.1. Electronic Searches

A comprehensive search for articles written in English was conducted in PubMed and the Cochrane Library. Studies were searched without time restrictions. Initial preliminary searches were carried out on 1 September 2024, followed by piloting the study selection process. A formal search was conducted on 24 October 2024. Search criteria were ((validation) AND (artificial intelligence)) AND (surgery), and restrictions were then added to include only title/abstract.

### 2.2. Study Selection

Inclusion criteria were original articles in English that included the use of Artificial Intelligence models in surgical medical specialties and that included details in the abstract or title about the clinical validation process used for the model. Within the Artificial Intelligence definition, articles were accepted when AI was referred to in the broadest terms, including references to machine learning, deep learning, neural networks, large language models, and specific names of known AI models. Exclusion criteria were non-English articles, review articles, articles that did not apply AI in the clinical setting of surgery, oral surgery studies, articles that did not refer to the validation process of the AI models being used, articles related to AI training, radiomics, and segmentation or development projects, as well as opinion, review or commentary articles that expressed experts’ opinions rather than contrasted original scientific work.

### 2.3. Outcomes

The primary outcome of this review was a comprehensive review of the uses of AI models in surgery, their risks and benefits, and a novel classification system named SURVAS (Surgical Validation Score), based on the different levels of evidence of each validation method.

### 2.4. Data Extraction and Management

A specific table was created for data extraction and was filled with data from the included studies. The following data was extracted: year of publication, journal, country of origin of first author, number of patients, size of dataset, availability of dataset, surgical specialty, relation to medical images, relation to cancer, operative stage, type of surgical use, type of validation, training data availability, benefits, risks and limitations, and ethical considerations.

### 2.5. Assessment of Risk of Bias in Included Studies

Risk of bias was assessed with the ROBINS-I tool (Risk Of Bias In Non-randomized Studies—of Interventions) [25]. As is standard according to ROBINS-I, the following biases were evaluated: confounding bias, participant selection, classification of interventions, deviations from intended interventions, missing data, measurement of outcomes, and selection of reported results. Each domain was judged with a “low risk”, “moderate risk”, or “high risk” of bias. The overall risk of bias for each study was then summarized by considering judgments across all domains, according to two researchers. Disagreement was resolved by the third author.

### 2.6. Measurement of Effect

The assessment items for each AI model were as follows: risks and limitations of the AI models referred to by authors, benefits of the use of AI in surgery, operative stage in which the model is applied, validation methods applied for the surgical AI model, and clinical use of the model. Other recorded study characteristics were the number of patients in which the model was applied, relation of the AI model to medical images, relation to cancer, multi- or single-centered, size and public availability of training data for the models, and discussion of ethical considerations in each study.

### 2.7. Data Analysis

The data were analyzed according to graphs and averages in an Excel chart including all categories for data extraction. Information was summed up with automatic Excel tools and manually revised. Data were analyzed by two researchers (A.M.V., N.K.), with inconsistencies resolved by a third researcher (J.M.E.). The study was a descriptive retrospective review, and no statistical methods or meta-analyses were conducted.

## 3. Results

### 3.1. Results of the Search

**Figure 1:**
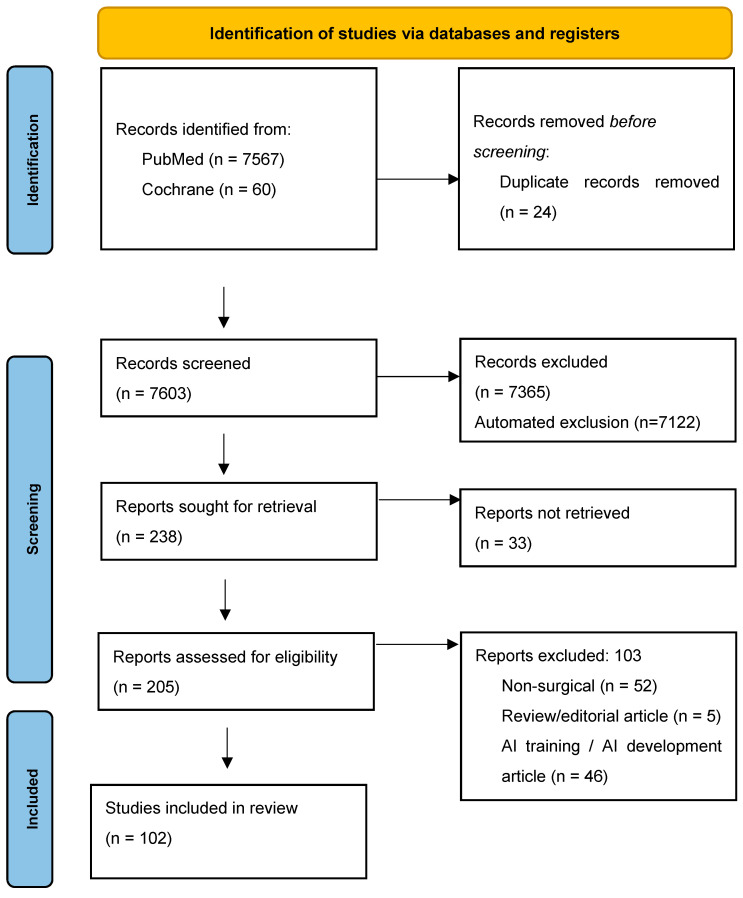
PRISMA flowchart.

The initial database search yielded a total of 7627 articles. After elimination of duplicates and screening of titles and abstracts, a total of 238 articles were sought for retrieval with a result of 205 obtained articles. After applying inclusion and exclusion criteria to the retrieved articles, a total of 102 articles were included in the study for data extraction, presenting a total of 2,837,211 patients. The PRISMA flowchart is presented in Figure 1.

### 3.2. Included Studies

After the application of screening and eligibility criteria, a total of 102 studies were included in the review. The included articles belonged to the following surgical specialties: orthopedics/spine surgery/neurosurgery [26,27,28,29,30,31,32,33,34,35,36,37,38,39,40,41,42,43,44,45,46,47,48,49,50,51,52,53,54,55,56,57,58,59,60], general surgery [61,62,63,64,65,66,67,68,69,70,71,72,73,74,75,76,77,78,79,80,81,82,83,84,85,86,87,88,89,90,91], anesthesiology [92,93,94,95,96,97,98], urology/gynecology [99,100,101,102,103,104], plastic surgery [105,106,107], ophthalmology [108,109,110,111], otolaryngology/head and neck surgery [112,113,114,115,116], and cardiac and thoracic surgery [117,118,119,120,121,122,123,124,125,126,127]. The included studies are summarized in Table 1, according to the order of appearance in search and data extraction.

### 3.3. Excluded Studies

From the 205 studies that were retrieved and assessed for eligibility, a total of 103 studies were excluded. The excluded studies were rejected for the following reasons: 52 studies were excluded due to not being a surgical application of AI models in clinical settings [129,130,131,132,133,134,135,136,137,138,139,140,141,142,143,144,145,146,147,148,149,150,151,152,153,154,155,156,157,158,159,160,161,162,163,164,165,166,167,168,169,170,171,172,173,174,175,176,177,178,179,180], 46 studies were excluded due to being considered as AI development, preclinical, experimental, or training studies [181,182,183,184,185,186,187,188,189,190,191,192,193,194,195,196,197,198,199,200,201,202,203,204,205,206,207,208,209,210,211,212,213,214,215,216,217,218,219,220,221,222,223,224,225,226], and 5 were excluded due to being considered review or editorial articles [15,227,228,229,230].

### 3.4. Characteristics of Results

A total of 2,837,211 patients were included in the study, with a dataset size of 18,137,987.

**Figure 2:**
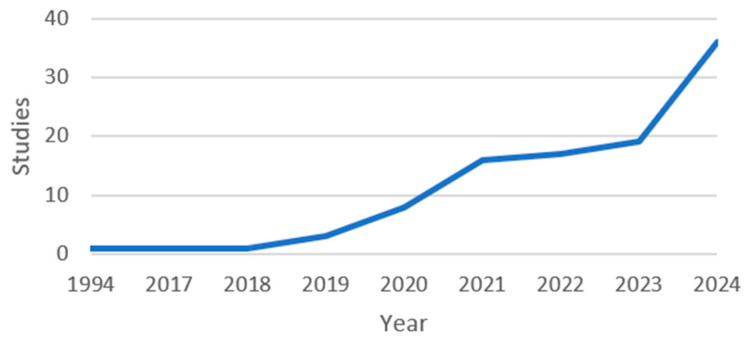
Publication year.

**Figure 3:**
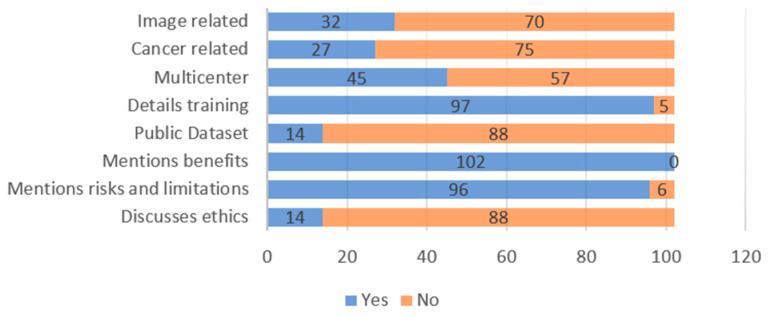
Key characteristics of included studies.

Studies according to publication year are shown in Figure 2, while the key characteristics of the included articles are shown in Figure 3.

### 3.5. Risk of Bias in Included Studies

The 102 included studies were assessed with the ROBINS-I tool, with the overall risk assessment presented in Table 1 (full information in Supplementary Material, Table S1). This study specifically indicated the inclusion criteria for articles containing assessment of validation of AI models, with a potential for selection bias. This means that the number of studies presenting AI models in surgery with no discussion of validation systems is unaccounted for, while the issue of validation is prone to be overly represented. This study cannot be used to assess the percentage of authors who disclose the validation systems used in their models. Publication bias is another risk, given that AI models with negative results are prone to non-publication.

### 3.6. Effect of the Intervention

**Figure 4:**
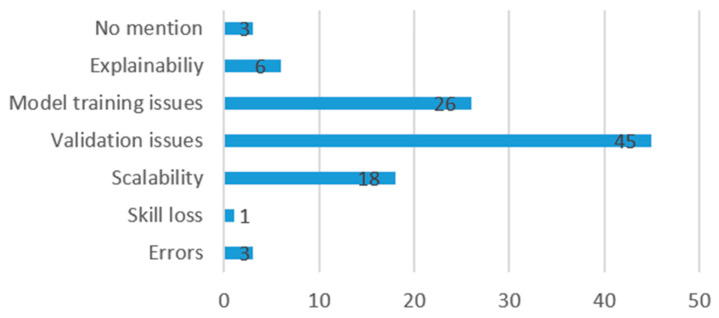
Limitations and risks of AI in surgery.

**Figure 5:**
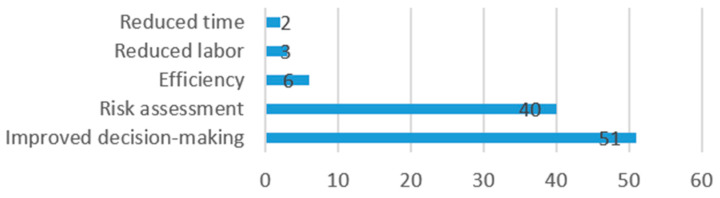
Benefits of AI in surgery.

**Figure 6:**
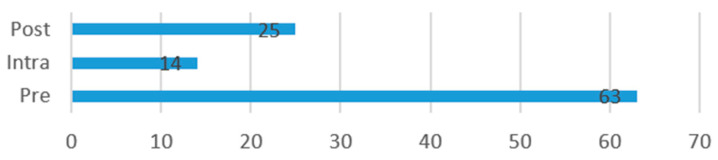
Operative Stage of AI Model Use.

**Figure 7:**
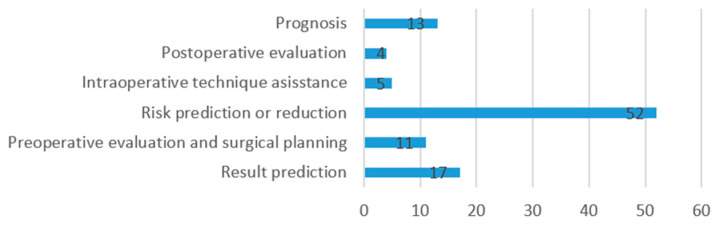
Uses of AI models in surgery.

After data extraction, the following information was compiled from each study: risks and limitations of the AI models referred to by authors (Figure 4), benefits of the use of the AI model in surgery (Figure 5), operative stage in which the model is applied (Figure 6), and clinical use of the model (Figure 7). 

**Figure 8:**
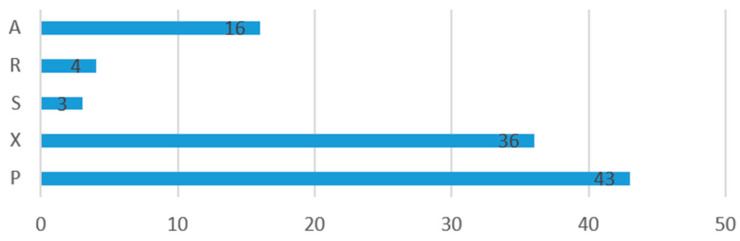
Articles grouped according to SURVAS validation method. (A) Advanced methods. (R) Resampling methods. (S) Single-split methods. (X) Cross-validation methods. (P) Performance metrics.

**Figure 9:**
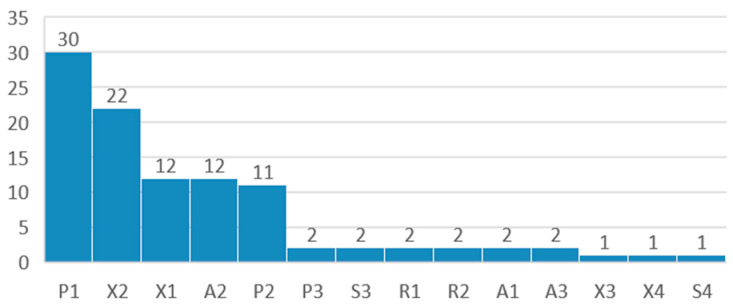
Articles grouped according to SURVAS validation subcategories.

A novel classification system for validation of AI models in surgery, named SURVAS (Surgical Validation Score) is presented in this work, and articles are distributed according to the evidence level of the validation methods used by authors, presented in Table 2, Table 3 and Table 4 and Figure 8 and Figure 9.

### 3.7. Narrative Analysis

The study included 102 articles resulting in a total of 2,837,211 patients. Included articles encompassed validated AI models in clinical use in surgery and obtained a total dataset size of 18,137,987. A rise in publications related to AI is seen beginning in the year 2019, with a sharp rise in the years 2023–2024. Out of the 102 articles, 27 (26%) were related to cancer and 32 (31%) were related to medical images. In addition, 45 (44%) studies were multicentered, while 57 (56%) were single centered. A total of 96 (94%) discussed limitations, risks, or disadvantages. Just 14 (14%) of the studies presented a public dataset. All the articles discussed the benefits of AI models in surgery, while only 14 (14%) studies discussed ethics related to AI. Finally, 97 (95%) articles discussed the training details.

Authors addressed the risks and limitations of their AI model as follows: 45 (44%) mentioned validation issues, 26 (26%) mentioned model training issues, 18 (18%) mentioned scalability issues, 6 (6%) mentioned explainability issues, 3 (3%) mentioned possibility of errors in the model, and 1 (1%) referred to risk of loss of skill. In addition, three (3%) did not refer to any risks or limitations in their models.

Authors described the benefits of use of their AI model as follows: 51 (50%) referred to improvements in the decision-making process, 40 (39%) referred to improvements in risk assessment, 6 (6%) referred to improved efficiency, and 3 (3%) referred to reduced labor, while 2 (2%) referred to reduced work time.

The operative stage of use of the AI model was as follows: 63 (62%) pre-operative, 25 (25%) post-operative, and 14 (14%) intra-operative use.

The uses of AI models were distributed as follows: 52 articles used (51%) AI models for risk prediction or reduction, 17 (17%) for prediction of outcomes, 13 (13%) for assessment of prognosis, 11 (11%) for pre-operative evaluation and surgical planning, and 5 (5%) for intra-operative technique assistance, while 4 articles (4%) used AI models for post-operative result or outcome evaluation.

### 3.8. SURVAS (Surgical Validation Score)

The Surgical Validation System (SURVAS) is a novel classification system, presented in Table 2, Table 3 and Table 4, while the distribution of the validation methods observed in this study according to SURVAS is presented in Figure 8 and Figure 9.

SURVAS divides the validation methods into two main sections, with subcategories, and an overall evidence level. The two major categories are Model Functionality Validation Methods (showing how the algorithm can be generalized) and Model Application Validation Methods (the model is applied to a practical situation, in a real environment). While the latter has only one category (advanced evaluations), the former is subdivided into four categories: (performance metrics, cross-validation methods, simple-split methods, and resampling and statistical methods). Within each category, the specific methods are listed in a scale of validity from 1 to 4, with 1 representing the highest validity, as detailed in Table 2.

According to this study, 43 (42%) of models were validated with performance metrics, 36 (35%) used cross-validation methods, 16 (16%) used advanced methods, 4 (4%) used resampling methods, and 3 (3%) used single-split methods. SURVAS subcategories for each study are represented in Figure 9. This study found that 46 (45%) of articles fall under high evidence categories, 47 (46%) fall under moderate evidence, and 7 (7%) fall under low evidence, while 2 (2%) fall under very low evidence.

## 4. Discussion

This study reviewed the use and validation systems for AI models applied to a total of 2,837,211 patients, coinciding with a sharp rise in publications regarding AI across healthcare in recent years [231]. The review yielded a novel classification system named SURVAS (Surgery Validation Score), which scored AI models according to their level of scientific evidence. According to this system, 55% of studies ranked validation methods as providing moderate evidence or lower, highlighting the need for progress in this area. Furthermore, 86% of the studies failed to provide publicly available datasets, evidencing the need for improved transparency. The predominant use of AI models was for risk assessment in the pre-operative setting, and the most cited benefit was improved decision-making.

A major issue related to AI in surgery is validation, which refers to the degree to which the AI model in use can be trusted to produce reliable results. A key feature of the studies is the fact that the overall quality of research on applications of AI is currently suboptimal, evidenced by the fact that most of the studies (56%) included were single-centered. Moreover, only 14% of authors used publicly available datasets for AI training, which has significant consequences. The quality and availability of the training datasets have a direct effect on the validation of AI models, a fact that did not go unnoticed by authors, as 44% mentioned validation issues and 26% mentioned model training issues as part of the risks and limitations in their work. Publicly available datasets create greater transparency in AI systems, and their lack is a known cause for concern [232]. The importance of data arises from the fact that AI models are trained on specific datasets that require high quality to ensure the accuracy of their predictions. Datasets can be public or private and vary in size. After the training phase, the models must be validated on a new dataset to ensure that their predictions are reliable. High quality public datasets contribute to safeguarding transparency in the validation process, as these datasets can be assessed by independent reviewers [233]. The low public availability of datasets observed in this review emphasizes the need for improvement in this area.

Validation has been shown to be a problematic issue in the field of AI [227,234]. As noted by Ho et al., a single positive validation score does not guarantee that the model can be generalized. According to this study, only 45% of studies were classified as having high levels of evidence according to SURVAS, leaving 55% as having moderate evidence or lower. The validation of AI models is relevant because it refers to the process of assessing the accuracy of results generated by these models, thereby helping clinicians trust the provided recommendations [235]. In the same way clinicians trust clinical studies in relation to medications, confirmation of the validation level of each AI model is necessary prior to clinical use with patients, highlighting the importance and practical use of classification systems such as SURVAS. There are different methods for the validation of AI models, with different levels of evidence [236]; however, in the clinical setting of surgery, few classifications systems are present.

SURVAS is divided into two main categories. The first, Model Functionality Validation, helps ensure that the model works well in theory—it accurately classifies or predicts outcomes based on test data. The second, Model Application Validation, goes a step further to ensure that the model works well in practice—it must be interpretable, usable, and reliable in real-world settings where decisions are made based on its predictions. Thus, it can be considered that the latter is a superior method. This classification is relevant because the results of this study show that only 45% of articles fall into high evidence categories, while the rest are moderate or lower. Furthermore, only 16% of articles applied advanced methods for real-world applications, emphasizing the low current confidence in the clinical use of AI in real-world scenarios, which is a need that has been previously noted in the literature [237]. As noted by Zhou et al. [238], ongoing monitoring and evaluation are necessary to maintain optimum quality, highlighting the relevance of standardized validation systems.

Another important issue reviewed in this study is the way AI is being used in surgery. According to this study, the uses of AI models were most predominantly (51%) risk prediction or reduction, followed by prediction of outcomes in 17% of the studies. Thirteen percent of uses were assessments of prognosis, while 11% were pre-operative evaluations and surgical planning. These results align with the current design of neural networks, which are built to predict results according to previous training datasets, and have been shown to surpass traditional statistical methods [239]. This fact also aligns well with the fact that 62% of studies used the AI model in the pre-operative setting. Only 5% of uses were intra-operative technique assistance, which means that currently, AI is not actively participating in surgical procedures; rather, it is a useful tool to predict outcomes in the broadest sense of the word, including risk and surgical results. The predictive power of AI and its role in preoperative evaluations have been well established [11]. However, the intra-operative application of AI is an interesting field, given that surgeons have been found to experience distractions and high prefrontal cortex demand during surgery, which can lead to potentially life-threatening complications [240]. AI models have been known to play key roles in crucial clinical tasks, including medical image analysis [241]. In this review, this potential is highlighted by the fact that 31% of surgical AI models were related to medical images. Another significant clinical use of AI is its application in cancer research [242]. The findings of this review indicate that 26% of AI model applications in the surgical setting were related to cancer, further emphasizing the valuable supporting role AI can play in this field.

According to the results, the most prevalent benefit of AI, mentioned by 50% of the authors, was improvement in the decision-making process. This result is in line with benefits discussed by Birkhoff et al., although exact data in the literature is limited [243]. This finding can be seen as a landmark in the introduction of AI into surgery, given the fact that decision making is a distinct human trait that carries enormous responsibility in medicine and is widely discussed in the field [244]. The fact that decision making is outlined as a major benefit for the use of AI foreshadows a paradigm shift in future generations, as AI models become increasingly more available, reliable, and efficient [245]. The second most cited benefit was the possibility of use in risk assessment [246], as stated in 40% of the studies in this review. While all the articles in this study discussed the benefits of AI models in surgery, only 14 (14%) studies discussed ethical issues related to AI, further emphasizing the need to understand and consider the long-term effects that AI can have on healthcare.

According to this study, current uses of AI models in surgery are predominantly in the pre-operative phase (62%), emphasizing the predictive capabilities of AI. This is further highlighted by the fact that 51% of studies employed AI models for risk prediction or reduction, and 16% used them for predicting results after surgery. The use of AI for predicting risk and complications is predominant in the literature, as reviewed by Bektas et al. [244]. AI and machine learning models have also shown advantages over conventional risk scores in cardiac surgery [247].

AI is a new field in healthcare, and therefore several issues and challenges arise. The rise of AI offers exciting uses, such as personalizing treatments [248] or having AI models participate in multidisciplinary teams [179]. AI has been successfully applied to recognize surgical steps using computer vision, promising help for training and improving surgical techniques [249]. An outstanding experimental use **of** AI models in surgery **is** an acoustic sensing device in orthopedic surgery, which can prevent surgical errors by detecting specific sounds of adverse bone drilling events [221]. However, the novelty of AI also offers challenges and limitations.

A major limitation of this review is the known heterogeneity of published literature on the subject [250], affecting the quality of the results. Sherbini et al. state that apart from this heterogeneity, published works often lack strong validation, further limiting the reliability of results [247]. As mentioned by Atiyeh et al., a major reason for these limitations is due to the fact that AI in healthcare is in its early life and high-quality evidence-based medicine regarding AI is currently scarce [251].

The primary limitations cited by authors are validation issues, noted in 45% of the studies included in this work. This limitation reinforces the importance of the SURVAS system presented herein. The presence of heterogeneity also stems from the large number of patients involved, which in turn supports the robustness of the study. The heterogeneity emerging from this study stresses how general guidelines can improve the quality of reporting, and authors are encouraged to follow guidelines such as CONSORT-AI and SPIRIT-AI [14]. Another limitation is the technical complexity of combining the healthcare sphere with problems relating to software engineering and development. Clear and transparent reporting of findings involves integrating and aligning medical and computer science concepts, which can create confusion, highlighting the relevance of AI literacy among stakeholders [252]. The lack of widespread regulations is another concern. As mentioned by Abbaker et al., robust regulatory frameworks can help ensure responsible AI implementation, taking into account ethical considerations [253]. There have been calls for improvement in scientific quality for AI research in surgery [254], emphasizing the need for further quality studies and comprehensive classification systems and guidelines. Furthermore, education for physicians on AI models is crucial for the safe and effective application of this new technology. Future research should focus on comprehensive guidelines for validation, while the real-time integration of machine learning into the operative stage is anticipated to be another key research path. Reliable and efficient AI integration currently appears to be a certain future for surgery, but this endeavor will require a multidimensional effort involving different stakeholders.

## 5. Conclusions

This review has assessed the uses and validation of AI models in 2,837,211 patients and presents a novel validation classification system named SURVAS (Surgery Validation Score). Validation methods were ranked as high evidence in only 45% of studies, while only 14% of the studies provided publicly available datasets. The predominant use of models was risk assessment, and the most predominant benefit was improved decision making. The most frequent use of AI was in the pre-operative setting. There is a need for a comprehensive validation classification method such as SURVAS for AI models in surgery.

## Figures and Tables

**Table 1:** Included studies. Risk of bias; Low: 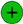; Moderate: 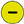; High: 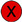.

Study	Year	Country	Journal	Patients	Dataset	Bias
Yu el al. [26]	2024	Republic of Korea	*Journal of Orthopaedic Surgery and Research*	714	500	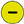
Jeon et al. [27]	2024	Republic of Korea	*BMC Musculoskeletal Disorders*	20	5,619,032	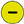
Li et al. [117]	2024	USA	*Journal of the American Heart Association*	35,214	35,214	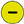
Altaf et al. [61]	2024	Pakistan	*Surgery*	192	192	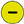
Zaidat et al. [28]	2024	USA	*Global Spine Journal*	535	535	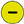
Tian et al. [118]	2024	China	*BMC Medical Informatics and Decision Making*	381	381	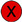
Jeong et al. [92]	2024	Republic of Korea	*Journal of Clinical Monitoring and Computing*	4754	4754	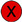
Sommer et al. [119]	2024	USA	*Frontiers in Artificial Intelligence*	591	591	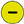
Chen et al. [112]	2022	Taiwan	*Diagnostics*	392	392	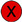
Katsuki et al. [120]	2021	Japan	*Surgical Neurology International*	140	140	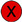
Park et al. [105]	2024	Republic of Korea	*Plastic and Reconstructive Surgery Global Open*	72	72	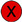
Lee et al. [62]	2024	Republic of Korea	*Surgical Endoscopy*	150	253,630	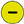
Aoyama et al. [63]	2024	Japan	*Surgical Endoscopy*	60	2771	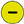
Hur el al. [121]	2024	Republic of Korea	*International Journal of Medical Informatics*	7843	7843	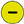
Wu et al. [93]	2024	China	*Bioengineering*	225	225	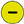
Yasin et al. [29]	2024	China	*European Journal of Medical Research*	580	580	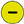
Florquin et al. [123]	2024	Belgium	*Journal of Anesthesia*	1364	1364	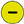
Barbieri et al. [122]	2024	Austria	*Journal of Clinical Medicine*	3595	3595	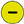
Yoshida et al. [64]	2024	Japan	*Langenbeck’s Archives of Surgery*	40	1,000,000	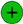
Kwon et al. [94]	2024	Republic of Korea	*Journal of Biomedical Informatics*	18,756	18,756	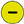
Dong et al. [124]	2024	United Kingdom	*JMIRx Med*	227,087	227,087	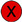
King et al. [30]	2024	Australia	*Cureus*	57	57	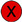
Benovic et al. [65]	2024	Germany	*Age and Ageing*	878	878	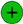
Lei et al. [31]	2024	China	*International Journal of Surgery*	52,707	52,707	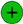
Dayan et al. [66]	2024	Israel	*Surgical Endoscopy*	499	499	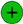
Namavarian et al. [113]	2024	Canada	*The Laryngoscope*	837	837	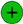
Yurick et al. [99]	2024	USA	*International Urogynecology Journal*	2049	2049	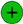
Bou-Nassif et al. [59]	2024	USA	*Communications Medicine*	40	32,051	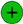
Bui et al. [32]	2024	Taiwan	*Bioengineering*	311	311	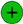
Cui et al. [33]	2024	China	*International Journal of Surgery*	-	455	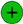
Wang et al. [67]	2024	China	*Head & Neck*	454	32,428	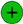
Peng et al. [34]	2024	China	*Frontiers in Neurology*	101	101	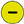
Dandurand et al. [35]	2024	Canada	*Global Spine Journal*	183	183	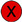
Abi-Rafeh et al. [106]	2024	Canada	*Aesthetic Plastic Surgery*	22	-	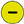
Nowakowska et al. [125]	2024	Poland	*Diagnostics*	224	224	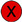
Ćirković et al. [108]	2023	Germany	*JMIR Formative Research*	100	-	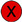
Turhan et al. [36]	2023	Turkey	*Clinics in Orthopedic Surgery*	313	313	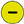
Kovoor et al. [68]	2023	Australia	*Surgery*	27,147	27,147	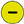
Kuo et al. [128]	2023	Taiwan	*Scientific Reports*	428	428	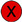
Bertsimas et al. [70]	2023	USA	*eClinicalMedicine*	951	951	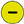
Shi et al. [37]	2024	China	*The Spine Journal*	276	276	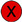
Saux et al. [71]	2023	France	*The Lancet Digital Health*	10,231	10,231	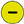
Matsuda et al. [72]	2023	Japan	*Annals of Surgical Oncology*	123	123	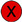
Mastropasqua et al. [115]	2024	Italy	*Graefe’s Archive for Clinical and Experimental Ophthalmology*	119	110	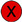
Zeitler et al. [116]	2024	USA	*Laryngoscope*	175	761	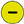
Hsu et al. [73]	2023	USA	*Surgical Endoscopy*	159,959	159,959	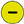
Kovoor et al. [74]	2023	Australia	*ANZ Journal of Surgery*	8826	42,572	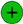
Kwong et al. [100]	2023	Canada	*The Lancet Digital Health*	2468	4936	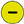
Chen et al. [75]	2023	USA	*American Surgeon*	262,923	262,923	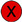
Flores-Balado et al. [38]	2023	Spain	*American Journal of Infection Control*	7444	19,661	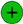
Wan et al. [109]	2023	China	*Ophthalmology and Therapy*	318	10,176	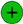
Huang et al. [107]	2023	Taiwan	*Plastic and Reconstructive Surgery*	176	805	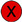
Chung et al. [76]	2023	Republic of Korea	*Journal of Cachexia, Sarcopenia and Muscle*	4615	4615	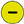
Shao et al. [101]	2023	Taiwan	*Journal of Personalized Medicine*	170	170	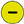
Laios et al. [102]	2022	United Kingdom	*Current Oncology*	285	1	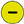
Choi et al. [95]	2022	Republic of Korea	*Journal of Clinical Medicine*	339,725	339,725	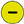
Röhr et al. [96]	2022	Germany	*Frontiers in Aging Neuroscience*	1355	1355	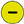
Yang et al. [39]	2022	China	*Frontiers in Public Health*	161	161	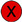
Chen et al. [77]	2022	United States	*Journal of Gastrointestinal Surgery*	213,827	213,827	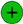
Khene et al. [103]	2023	France	*European Urology Oncology*	4067	4067	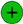
Lee et al. [97]	2022	Republic of Korea	*npj Digital Medicine*	454,404	454,404	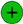
Yossofzai et al. [40]	2022	Canada	*Epilepsia*	801	801	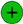
Valliani et al. [41]	2022	United States	*World Neurosurgery*	497,536	497,536	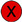
Bertsimas et al. [78]	2022	United States	*JAMA Surgery*	1843	1843	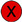
Deng et al. [79]	2022	United States	*JAMA Network Open*	2372	2372	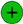
Scherer et al. [42]	2022	Germany	*The Spine Journal*	179	179	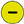
van de Sande et al. [80]	2022	The Netherlands	*Surgery*	2035	2447	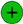
Ichimasa et al. [81]	2022	Japan	*Gastrointestinal Endoscopy*	511	511	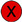
Karhade et al. [43]	2022	USA	*Clinical Orthopaedics and Related Research*	3223	-	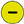
Hinterwimmer et al. [44]	2023	Germany	*Knee Surgery, Sports Traumatology, Arthroscopy*	864	864	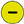
Zhang et al. [45]	2021	Singapore	*Arthroplasty*	1508	1508	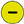
Ory et al. [104]	2022	USA	*World Journal of Men’s Health*	240	-	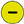
Martin et al. [46]	2022	USA	*Knee Surgery, Sports Traumatology, Arthroscopy*	10,922	-	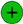
Lo Muzio et al. [126]	2021	Italy	*Journal of Clinical Medicine*	12	86	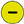
Mazaki et al. [82]	2021	Japan	*Anticancer Research*	256	256	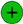
Kunze et al. [47]	2021	USA	*The Orthopaedic Journal of Sports Medicine*	442	442	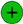
Kamaleswaran et al. [83]	2021	United States	*Frontiers in Physiology*	5748	8,350,000	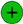
Cole et al. [84]	2021	United States	*Journal of Surgical Research*	93,024	93,024	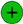
Cao et al. [85]	2021	Sweden	*JMIR Medical Informatics*	8057	8057	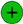
Ji et al. [86]	2021	China	*Journal of Hepatocellular Carcinoma*	2778	2778	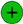
Tanikawa et al. [114]	2021	Japan	*Scientific Reports*	137	137	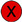
Zhao et al. [48]	2021	China	*Frontiers in Surgery*	245	245	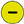
Bang et al. [87]	2021	Republic of Korea	*Journal of Medical Internet Research*	2703	2703	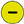
Wissel et al. [49]	2021	USA	*Acta Neurologica Scandinavica*	13,484	13,484	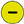
El Hechi et al. [88]	2021	USA	*Journal of the American College of Surgeons*	78,880	78,880	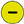
Maurer et al. [89]	2023	USA	*Annals of Surgery*	29,366	-	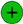
Wirries et al. [50]	2021	Germany	*European Spine Journal*	60	60	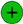
Penny-Dimri et al. [127]	2020	Australia	*Seminars in Thoracic and Cardiovascular Surgery*	96,653	97,964	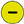
Yoo et al. [110]	2020	Republic of Korea	*Translational Vision Science & Technology*	1848	1848	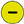
Carmona González et al. [111]	2021	Spain	*Eye*	260	260	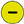
Fatima et al. [51]	2020	USA	*World Neurosurgery*	80,610	80,610	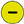
Karhade et al. [60]	2019	USA	*The Spine Journal*	1053	1053	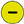
Karhade et al. [52]	2020	USA	*The Spine Journal*	5860	5860	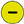
Hopkins et al. [53]	2020	USA	*Clinical Neurology and Neurosurgery*	4046	4046	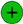
Karhade et al. [54]	2020	USA	*The Spine Journal*	1000	1000	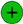
Hopkins et al. [55]	2020	USA	*Journal of Neurosurgery: Spine*	23,264	23,264	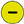
Stopa et al. [56]	2019	USA	*Journal of Neurosurgery: Spine*	144	144	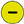
Jo et al. [57]	2020	Republic of Korea	*Knee Surgery, Sports Traumatology, Arthroscopy*	2086	2086	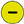
He et al. [58]	2019	China	*European Radiology*	56	56	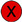
Ichimasa et al. [90]	2018	Japan	*Endoscopy*	690	690	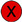
Sammour et al. [91]	2017	Australia	*Techniques in Coloproctology*	402	402	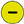
Lette et al. [98]	1994	Canada	*Clinical Cardiology*	360	360	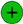

**Table 2:** Levels of evidence; 1: High evidence, 2: moderate evidence, 3: low evidence, 4: very low evidence.

Evidence Level	Methods	SURVAS
Level 1 (High Evidence)	Widely accepted methods in model validation, with high statistical robustness and commonly used for testing generalizable models (e.g., AUROC, repeated cross-validation, Cox regression).	P1, X1, R1, A1
Level 2 (Moderate Evidence)	Robust methods, but less generalized or specifically used in certain clinical or research contexts (e.g., F1-score, simple bootstrap).	P2, X2, R2, S2, A2
Level 3 (Low Evidence)	Frequently used methods that do not always provide the best statistical evaluation in all contexts, or may be complementary (e.g., 4-fold cross-validation, 70–20-10 partition).	P3, X3, S3, A3
Level 3 (Very Low Evidence)	Methods that offer limited value and are mainly used in exploratory or smaller studies, or experimental phase (e.g., hold-out validation in specific contexts)	A2

**Table 3:** Model Functionality Validation in Surgical Validation Score (SURVAS).

Category	Validation Method	Evidence Level	SURVAS
Performance Metrics (P)	Concordance Index (c-index) and Brier Score	1	P1
	ROC and AUC	1	P1
	C-Statistic	1	P1
	Harrell, Brier, Calibration, Hosmer–Lemeshow	1	P1
	Cohen’s κ Coefficient, Confusion Matrix	2	P2
	F1-Score	2	P2
	Sensitivity, Specificity, Diagnostic Certainty	2	P2
	Dice Coefficient	2	P2
	MAD, RMSE, and Bland–Altman Plots	3	P3
Cross-Validation Methods (X)	5-Fold Cross-Validation Repeated 20 Times	1	X1
	10-Fold Cross-Validation	1	X1
	10-Repeated 5-Fold Cross-Validation	1	X1
	Stratified 10-Fold Cross-Validation	1	X1
	15-Fold Cross-Validation	2	X2
	K-Fold Cross-Validation	2	X2
	5-Fold Cross-Validation	2	X2
	11-Fold Cross-Validation	3	X3
	4-Fold Cross-Validation	4	X4
Simple Split Methods (S)	Train-Test-Validation Split of 70–20-10	3	S3
	80% Training, 20% Testing	3	S3
	Hold-Out Validation, 30/10 Videos	4	S4
Resampling and Statistical Methods (R)	DeLong Testing for AUROC	1	R1
	Independent External Validation	1	R1
	Wilcoxon Signed-Rank, Paired *t*-test	2	R2
	Bootstrap Method	2	R2

**Table 4:** Model Application Validation in Surgical Validation Score (SURVAS).

Category	Validation Method	Evidence Level	SURVAS
Advanced Evaluations (A)	Multi-Tree XGBoost with 5-fold Cross-Validation	1	A1
	ROC Curves and Cox Regression Analysis	1	A1
	SHAP Interpretation, ROC Curves, Precision-Recall Metrics	2	A2
	10-Fold Cross-Validation with FROC Curve	2	A2
	AUC, Shapley Additive Explanations (SHAP)	2	A2
	ROC Curve, MCC, AUC Metrics	2	A2
	Likert Scale	3	A3

## Data Availability

The original contributions presented in this study are included in the article/Supplementary Materials. Further inquiries can be directed to the corresponding author.

## Supplementary Materials

The following supporting information can be downloaded at: https://www.mdpi.com/article/10.3390/jcm13237108/s1, Table S1: ROBINS-I.
