# Supplementary material for: Artificial Intelligence in Surgery: A Systematic Review of Use and Validation
Source: J Clin Med. 2024 Nov 24;13(23):7108. doi: 10.3390/jcm13237108 (PMC11642125; doi:10.3390/jcm13237108)
Supplement: Supplementary file 1 [file jcm-13-07108-s001.zip › jcm-3314790-supplementary.pdf]

Study	D1	D2	D3	D4	D5	D6	D7
Development of an artificial intelligence model for predicting implant size in total knee arthroplasty using simple X-ray images.<br>Yu Y, Cho YJ, Park S, Kim YH, Goh TS.<br>J Orthop Surg Res. 2024 Aug 27;19(1):516. doi: 10.1186/s13018-024-05013-2.<br>PMID: 39192371	2	2	1	1	2	2	1
Clinical validation of artificial intelligence-based preoperative virtual reduction for Neer 3- or 4-part proximal humerus fractures.<br>Jeon YD, Jung KH, Kim MS, Kim H, Yoon DK, Park KB.<br>BMC Musculoskelet Disord. 2024 Aug 27;25(1):669. doi: 10.1186/s12891-024-07798-z.<br>PMID: 39192203	2	1	2	1	2	1	2
Using Machine Learning to Predict Outcomes Following Transfemoral Carotid Artery Stenting.<br>Li B, Eisenberg N, Beaton D, Lee DS, Al-Omran L, Wijeysundera DN, Hussain MA, Rotstein OD, de Mestral C, Mamdani M, Roche-Nagle G, Al-Omran M.<br>J Am Heart Assoc. 2024 Aug 27:e035425. doi: 10.1161/JAHA.124.035425. Online ahead of print.<br>PMID: 39189482 Free article.	2	1	2	1	1	1	2
Artificial intelligence-based model for the recurrence of hepatocellular carcinoma after liver transplantation.<br>Altaf A, Mustafa A, Dar A, Nazer R, Riyaz S, Rana A, Bhatti ABH.<br>Surgery. 2024 Aug 23:S0039-6060(24)00558-0. doi: 10.1016/j.surg.2024.07.039. Online ahead of print.<br>PMID: 39181726	2	1	2	1	2	1	1
Explainable Machine Learning Approach to Prediction of Prolonged Intesive Care Unit Stay in Adult Spinal Deformity Patients: Machine Learning Outperforms Logistic Regression.<br>Zaidat B, Kurapatti M, Gal JS, Cho SK, Kim JS.<br>Global Spine J. 2024 Aug 21:21925682241277771. doi: 10.1177/21925682241277771. Online ahead of print.<br>PMID: 39169510 Free article.	2	1	1	2	2	1	2

Machine learning model predicts airway stenosis requiring clinical intervention in patients after lung transplantation: a retrospective case-controlled study.<br>Tian D, Zuo YJ, Yan HJ, Huang H, Liu MZ, Yang H, Zhao J, Shi LZ, Chen JY.<br>BMC Med Inform Decis Mak. 2024 Aug 19;24(1):229. doi: 10.1186/s12911-024-02635-8.<br>PMID: 39160522 Free PMC article.	3	1	2	1	2	2	2
Prediction of intraoperative hypotension using deep learning models based on non-invasive monitoring devices.<br>Jeong H, Kim D, Kim DW, Baek S, Lee HC, Kim Y, Ahn HJ.<br>J Clin Monit Comput. 2024 Aug 19. doi: 10.1007/s10877-024-01206-6. Online ahead of print.<br>PMID: 39158783	3	1	1	2	2	1	2
Deep learning for prediction of post-thrombectomy outcomes based on admission CT angiography in large vessel occlusion stroke.<br>Sommer J, Dierksen F, Zeevi T, Tran AT, Avery EW, Mak A, Malhotra A, Matouk CC, Falcone GJ, Torres-Lopez V, Aneja S, Duncan J, Sansing LH, Sheth KN, Payabvash S.<br>Front Artif Intell. 2024 Aug 1;7:1369702. doi: 10.3389/frai.2024.1369702. eCollection 2024.<br>PMID: 39149161 Free PMC article.	2	1	2	2	1	2	2
Deep Learning Artificial Intelligence to Predict the Need for Tracheostomy in Patients of Deep Neck Infection Based on Clinical and Computed Tomography Findings—Preliminary Data and a Pilot Study<br>S-L Chen, S-C Chin, C-Y Ho<br>Diagnostics, 2022, 12(8) | added to CENTRAL: 31 October 2022 | 2022 Issue 10	2	2	2	2	2	3	3
Postsurgical functional outcome prediction model using deep learning framework (Prediction One, Sony Network Communications Inc.) for hypertensive intracerebral hemorrhage<br>M Katsuki, Y Kakizawa, A Nishikawa, Y Yamamoto, T Uchiyama<br>Surgical neurology international, 2021, 12 | added to CENTRAL: 30 June 2021 | 2021 Issue 06	3	2	2	2	2	3	3

A Comprehensive Assessment of Soft-tissue Sagging after Zygoma Reduction Surgery through Artificial Intelligence Analysis.<br>Park YY, Kim KK, Park B.<br>Plast Reconstr Surg Glob Open. 2024 Aug 13;12(8):e6055. doi: 10.1097/GOX.0000000000006055. eCollection 2024 Aug.<br>PMID: 39139838 Free PMC article.	2	2	1	2	2	2	3
Intraoperative detection of parathyroid glands using artificial intelligence: optimizing medical image training with data augmentation methods.<br>Lee JH, Ku E, Chung YS, Kim YJ, Kim KG.<br>Surg Endosc. 2024 Aug 13. doi: 10.1007/s00464-024-11115-z. Online ahead of print.<br>PMID: 39138679	1	2	1	3	1	2	2
Artificial intelligence for surgical safety during laparoscopic gastrectomy for gastric cancer: Indication of anatomical landmarks related to postoperative pancreatic fistula using deep learning.<br>Aoyama Y, Matsunobu Y, Etoh T, Suzuki K, Fujita S, Aiba T, Fujishima H, Empuku S, Kono Y, Endo Y, Ueda Y, Shiroshita H, Kamiyama T, Sugita T, Morishima K, Ebe K, Tokuyasu T, Inomata M.<br>Surg Endosc. 2024 Aug 2. doi: 10.1007/s00464-024-11117-x. Online ahead of print.<br>PMID: 39093411	2	1	1	2	1	2	2
Development, validation, and usability evaluation of machine learning algorithms for predicting personalized red blood cell demand among thoracic surgery patients.<br>Hur S, Yoo J, Min JY, Jeon YJ, Cho JH, Seo JY, Cho D, Kim K, Lee Y, Cha WC.<br>Int J Med Inform. 2024 Jul 18;191:105543. doi: 10.1016/j.ijmedinf.2024.105543. Online ahead of print.<br>PMID: 39084087 Free article.	2	1	1	3	1	2	2
Difficult Airway Assessment Based on Multi-View Metric Learning.<br>Wu J, Yao Y, Zhang G, Li X, Peng B.<br>Bioengineering (Basel). 2024 Jul 11;11(7):703. doi: 10.3390/bioengineering11070703.<br>PMID: 39061785 Free PMC article.	2	1	1	2	1	2	2

Machine learning-enabled prediction of prolonged length of stay in hospital after surgery for tuberculosis spondylitis patients with unbalanced data: a novel approach using explainable artificial intelligence (XAI).<br>Yasin P, Yimit Y, Cai X, Aimaiti A, Sheng W, Mamat M, Nijiati M.<br>Eur J Med Res. 2024 Jul 25;29(1):383. doi: 10.1186/s40001-024-01988-0.<br>PMID: 39054495	2	1	1	2	1	3	2
Pediatric cardiac surgery: machine learning models for postoperative complication prediction.<br>Florquin R, Florquin R, Schmartz D, Dony P, Briganti G.<br>J Anesth. 2024 Jul 19. doi: 10.1007/s00540-024-03377-7. Online ahead of print.<br>PMID: 39028323	2	1	1	2	2	2	2
A Neuronal Network-Based Score Predicting Survival in Patients Undergoing Aortic Valve Intervention: The ABC-AS Score.<br>Barbieri F, Pfeifer BE, Senoner T, Dobner S, Spitaler P, Semsroth S, Lambert T, Zweiker D, Neururer SB, Scherr D, Schmidt A, Feuchtner GM, Hoppe UC, Adukauskaite A, Reinthaler M, Landmesser U, Müller S, Steinwender C, Dichtl W.<br>J Clin Med. 2024 Jun 25;13(13):3691. doi: 10.3390/jcm13133691.<br>PMID: 38999259	2	1	1	2	1	2	2
Surgical step recognition in laparoscopic distal gastrectomy using artificial intelligence: a proof-of-concept study.<br>Yoshida M, Kitaguchi D, Takeshita N, Matsuzaki H, Ishikawa Y, Yura M, Akimoto T, Kinoshita T, Ito M.<br>Langenbecks Arch Surg. 2024 Jul 12;409(1):213. doi: 10.1007/s00423-024-03411-y.<br>PMID: 38995411	2	1	1	1	1	1	1
Non-invasive prediction of massive transfusion during surgery using intraoperative hemodynamic monitoring data.<br>Kwon D, Mi Jung Y, Lee HC, Kyong Kim T, Kim K, Lee G, Kim D, Lee SB, Mi Lee S.<br>J Biomed Inform. 2024 Aug;156:104680. doi: 10.1016/j.jbi.2024.104680. Epub 2024 Jun 22.<br>PMID: 38914411	2	1	1	1	1	1	2

Performance Drift in Machine Learning Models for Cardiac Surgery Risk Prediction: Retrospective Analysis.<br>Dong T, Sinha S, Zhai B, Fudulu D, Chan J, Narayan P, Judge A, Caputo M, Dimagli A, Benedetto U, Angelini GD.<br>JMIRx Med. 2024 Jun 12;5:e45973. doi: 10.2196/45973.<br>PMID: 38889069	2	3	2	3	2	2	2
Use of Artificial Intelligence in the Prediction of Chiari Malformation Type 1 Recurrence After Posterior Fossa Decompressive Surgery.<br>King V, Liu S, Russo C, Jayasekara M, Stoodley M, Di Ieva A.<br>Cureus. 2024 May 22;16(5):e60879. doi: 10.7759/cureus.60879. eCollection 2024 May.<br>PMID: 38784688	2	3	1	2	2	2	2
Introducing a machine learning algorithm for delirium prediction-the Supporting SURgery with GEriatric Co-Management and AI project (SURGE-Ahead).<br>Benovic S, Ajlani AH, Leinert C, Fotteler M, Wolf D, Steger F, Kestler H, Dallmeier D, Denkinger M, Eschweiler GW, Thomas C, Kocar TD.<br>Age Ageing. 2024 May 1;53(5):afae101. doi: 10.1093/ageing/afae101.<br>PMID: 38776213	2	1	1	2	1	2	1
Establishment and validation of an artificial intelligence web application for predicting postoperative in-hospital mortality in patients with hip fracture: a national cohort study of 52 707 cases.<br>Lei M, Feng T, Chen M, Shen J, Liu J, Chang F, Chen J, Sun X, Mao Z, Li Y, Yin P, Tang P, Zhang L.<br>Int J Surg. 2024 Aug 1;110(8):4876-4892. doi: 10.1097/JS9.0000000000001599.<br>PMID: 38752505	2	1	1	2	1	2	1
Implementation of artificial intelligence-based computer vision model in laparoscopic appendectomy: validation, reliability, and clinical correlation.<br>Dayan D, Dvir N, Agbariya H, Nizri E.<br>Surg Endosc. 2024 Jun;38(6):3310-3319. doi: 10.1007/s00464-024-10847-2. Epub 2024 Apr 25.<br>PMID: 38664295	2	1	1	2	1	2	1

Length of Stay Prediction Models for Oral Cancer Surgery: Machine Learning, Statistical and ACS-NSQIP.<br>Namavarian A, Gabinet-Equihua A, Deng Y, Khalid S, Ziai H, Deutsch K, Huang J, Gilbert RW, Goldstein DP, Yao CMKL, Irish JC, Enepekides DJ, Higgins KM, Rudzicz F, Eskander A, Xu W, de Almeida JR.<br>Laryngoscope. 2024 Aug;134(8):3664-3672. doi: 10.1002/lary.31443. Epub 2024 Apr 23.<br>PMID: 38651539	2	1	1	2	1	2	1
Prediction of Postoperative Urinary Tract Infection Following Benign Gynecologic Surgery.<br>Yurick S, Ray S, El-Nashar S, Brennand E, Kim-Fine S, Sanaee M, Regan S, Geoffrion R, Occhino J, Hijaz A, Sheyn D.	2	1	1	1	2	1	1
Development and prospective validation of an artificial intelligence-based smartphone app for rapid intraoperative pituitary adenoma identification.<br>Bou-Nassif R, Reiner AS, Pease M, Bale T, Cohen MA, Rosenblum M, Tabar V.<br>Commun Med (Lond). 2024 Mar 13;4(1):45. doi: 10.1038/s43856-024-00469-z.<br>PMID: 38480833	2	2	1	1	1	1	2
Development of End-to-End Artificial Intelligence Models for Surgical Planning in Transforaminal Lumbar Interbody Fusion.<br>Bui AT, Le H, Hoang TT, Trinh GM, Shao HC, Tsai PI, Chen KJ, Hsieh KL, Huang EW, Hsu CC, Mathew M, Lee CY, Wang PY, Huang TJ, Wu MH.<br>Bioengineering (Basel). 2024 Feb 8;11(2):164. doi: 10.3390/bioengineering11020164.<br>PMID: 38391650	2	2	1	1	1	2	2
Establishment and validation of an interactive artificial intelligence platform to predict postoperative ambulatory status for patients with metastatic spinal disease: a multicenter analysis.<br>Cui Y, Shi X, Qin Y, Wang Q, Cao X, Che X, Pan Y, Wang B, Lei M, Liu Y.<br>Int J Surg. 2024 May 1;110(5):2738-2756. doi: 10.1097/JS9.0000000000001169.<br>PMID: 38376838 Free	2	1	1	2	1	1	1
Intraoperative AI-assisted early prediction of parathyroid and ischemia alert in endoscopic thyroid surgery.<br>Wang B, Yu JF, Lin SY, Li YJ, Huang WY, Yan SY, Wang SS, Zhang LY, Cai SJ, Wu SB, Li MY, Wang TY, Abdelhamid Ahmed AH, Randolph GW, Chen F, Zhao WX.<br>Head Neck. 2024 Aug;46(8):1975-1987. doi: 10.1002/hed.27629. Epub 2024 Feb	2	1	1	2	1	1	1

13.<br>PMID: 38348564							
Deep learning and machine learning predictive models for neurological function after interventional embolization of intracranial aneurysms.<br>Peng Y, Wang Y, Wen Z, Xiang H, Guo L, Su L, He Y, Pang H, Zhou P, Zhan X.<br>Front Neurol. 2024 Jan 24;15:1321923. doi: 10.3389/fneur.2024.1321923. eCollection 2024.<br>PMID: 38327618	2	1	1	2	1	2	2
Predictive Algorithm for Surgery Recommendation in Thoracolumbar Burst Fractures Without Neurological Deficits.<br>Dandurand C, Fallah N, Öner CF, Bransford RJ, Schnake K, Vaccaro AR, Benneker LM, Vialle E, Schroeder GD, Rajasekaran S, El-Skarkawi M, Kanna RM, Aly M, Holas M, Canseco JA, Muijs S, Popescu EC, Tee JW, Camino-Willhuber G, Joaquim AF, Keynan O, Chhabra HS, Bigdon S, Spiegel U, Dvorak MF.<br>Global Spine J. 2024 Feb;14(1_suppl):56S-61S. doi: 10.1177/21925682231203491.<br>PMID: 38324597	3	2	2	2	2	3	3
Complications Following Body Contouring: Performance Validation of Bard, a Novel AI Large Language Model, in Triaging and Managing Postoperative Patient Concerns.<br>Abi-Rafeh J, Mroueh VJ, Bassiri-Tehrani B, Marks J, Kazan R, Nahai F.<br>Aesthetic Plast Surg. 2024 Mar;48(5):953-976. doi: 10.1007/s00266-023-03819-9. Epub 2024 Jan 25.<br>PMID: 38273152	2	2	1	1	2	2	2
AI-Enhanced Predictive Modeling for Identifying Depression and Delirium in Cardiovascular Patients Scheduled for Cardiac Surgery.<br>Nowakowska K, Sakellarios A, Kaźmierski J, Fotiadis DI, Pezoulas VC.<br>Diagnostics (Basel). 2023 Dec 27;14(1):67. doi: 10.3390/diagnostics14010067.<br>PMID: 38201376	3	2	1	2	3	3	3
Exploring the Potential of ChatGPT-4 in Predicting Refractive Surgery Categorizations: Comparative Study.<br>Ćirković A, Katz T.<br>JMIR Form Res. 2023 Dec 28;7:e51798. doi:	3	2	1	2	3	3	2

10.2196/51798.<br>PMID: 38153777							
Predicting Prolonged Wound Drainage after Hemiarthroplasty for Hip Fractures: A Stacked Machine Learning Study.<br>Turhan S, Canbek U, Dubektas-Canbek T, Dogu E.<br>Clin Orthop Surg. 2023 Dec;15(6):894-901. doi: 10.4055/cios22181. Epub 2023 Oct 20.<br>PMID: 38045590	2	1	1	1	2	1	2
Surgery's Rosetta Stone: Natural language processing to predict discharge and readmission after general surgery.<br>Kovoor JG, Bacchi S, Gupta AK, Stretton B, Nann SD, Aujayeb N, Lu A, Nathin K, Lam L, Jiang M, Lee S, To MS, Ovenden CD, Hewitt JN, Goh R, Gluck S, Reid JL, Khurana S, Dobbins C, Hewett PJ, Padbury RT, Malycha J, Trochsler MI, Hugh TJ, Maddern GJ.<br>Surgery. 2023 Dec;174(6):1309-1314. doi: 10.1016/j.surg.2023.08.021. Epub 2023 Sep 29.<br>PMID: 37778968	2	1	1	1	2	1	2
Artificial intelligence based system for predicting permanent stoma after sphincter saving operations.<br>Kuo CY, Kuo LJ, Lin YK.<br>Sci Rep. 2023 Sep 25;13(1):16039. doi: 10.1038/s41598-023-43211-w.<br>PMID: 37749194	3	2	1	3	3	1	2
An interpretable AI model for recurrence prediction after surgery in gastrointestinal stromal tumour: an observational cohort study.<br>Bertsimas D, Margonis GA, Tang S, Koulouras A, Antonescu CR, Brennan MF, Martin-Broto J, Rutkowski P, Stasinos G, Wang J, Pikoulis E, Bylina E, Sobczuk P, Gutierrez A, Jadeja B, Tap WD, Chi P, Singer S.<br>EClinicalMedicine. 2023 Sep 9;64:102200. doi: 10.1016/j.eclinm.2023.102200. eCollection 2023 Oct.<br>PMID: 37731933	3	2	1	2	1	1	2
Development and validation of a web-based artificial intelligence prediction model to assess massive intraoperative blood loss for metastatic spinal disease using machine learning techniques.<br>Shi X, Cui Y, Wang S, Pan Y, Wang B, Lei M.<br>Spine J. 2024 Jan;24(1):146-160. doi: 10.1016/j.spinee.2023.09.001. Epub 2023	3	2	1	3	1	2	2

Sep 11.<br>PMID: 37704048							
Development and validation of an interpretable machine learning-based calculator for predicting 5-year weight trajectories after bariatric surgery: a multinational retrospective cohort SOPHIA study.<br>Saux P, Bauvin P, Raverdy V, Teigny J, Verkindt H, Soumphonphakdy T, Debert M, Jacobs A, Jacobs D, Monpellier V, Lee PC, Lim CH, Andersson-Assarsson JC, Carlsson L, Svensson PA, Galtier F, Dezfoulian G, Moldovanu M, Andrieux S, Couster J, Lepage M, Lembo E, Verrastro O, Robert M, Salminen P, Mingrone G, Peterli R, Cohen RV, Zerrweck C, Nocca D, Le Roux CW, Caiazzo R, Preux P, Pattou F.<br>Lancet Digit Health. 2023 Oct;5(10):e692-e702. doi: 10.1016/S2589-7500(23)00135-8. Epub 2023 Aug 29.<br>PMID: 37652841	2	1	1	2	2	1	1
Endoscopic Evaluation of Pathological Complete Response Using Deep Neural Network in Esophageal Cancer Patients Who Received Neoadjuvant Chemotherapy-Multicenter Retrospective Study from Four Japanese Esophageal Centers.<br>Matsuda S, Irino T, Okamura A, Mayanagi S, Booka E, Takeuchi M, Kawakubo H, Takeuchi H, Watanabe M, Kitagawa Y.<br>Ann Surg Oncol. 2023 Nov;30(12):7472-7480. doi: 10.1245/s10434-023-13862-0. Epub 2023 Aug 5.<br>PMID: 37543555	3	2	1	3	2	1	2
A deep learning approach to investigate the filtration bleb functionality after glaucoma surgery: a preliminary study.<br>Mastropasqua L, Agnifili L, Brescia L, Figus M, Posarelli C, Oddone F, Giammaria S, Sacchi M, Pavan M, Innocenti DD, Olivotto V, Sensi SL, Mastropasqua R.<br>Graefes Arch Clin Exp Ophthalmol. 2024 Jan;262(1):149-160. doi: 10.1007/s00417-023-06170-6. Epub 2023 Aug 2.<br>PMID: 37530849	3	2	1	3	2	2	1

Predicting Acoustic Hearing Preservation Following Cochlear Implant Surgery Using Machine Learning.<br>Zeitler DM, Buchlak QD, Ramasundara S, Farrokhi F, Esmaili N.<br>Laryngoscope. 2024 Feb;134(2):926-936. doi: 10.1002/lary.30894. Epub 2023 Jul 14.<br>PMID: 37449725	2	2	1	1	2	1	1
Application of machine learning to predict postoperative gastrointestinal bleed in bariatric surgery.<br>Hsu JL, Chen KA, Butler LR, Bahraini A, Kapadia MR, Gomez SM, Farrell TM.<br>Surg Endosc. 2023 Sep;37(9):7121-7127. doi: 10.1007/s00464-023-10156-0. Epub 2023 Jun 13.<br>PMID: 37311893	2	1	2	1	2	1	1
The Adelaide Score: An artificial intelligence measure of readiness for discharge after general surgery.<br>Kovoor JG, Bacchi S, Gupta AK, Stretton B, Malycha J, Reddi BA, Liew D, O'Callaghan PG, Beltrame JF, Zannettino AC, Jones KL, Horowitz M, Dobbins C, Hewett PJ, Trochsler MI, Maddern GJ.<br>ANZ J Surg. 2023 Sep;93(9):2119-2124. doi: 10.1111/ans.18546. Epub 2023 Jun 1.<br>PMID: 37264548	2	1	1	1	2	1	1
Development, multi-institutional external validation, and algorithmic audit of an artificial intelligence-based Side-specific Extra-Prostatic Extension Risk Assessment tool (SEPERA) for patients undergoing radical prostatectomy: a retrospective cohort study.<br>Kwong JCC, Khondker A, Meng E, Taylor N, Kuk C, Perlis N, Kulkarni GS, Hamilton RJ, Fleshner NE, Finelli A, van der Kwast TH, Ali A, Jamal M, Papanikolaou F, Short T, Srigley JR, Colinet V, Peltier A, Diamand R, Lefebvre Y, Mandoorah Q, Sanchez-Salas R, Macek P, Cathelineau X, Eklund M, Johnson AEW, Feifer A, Zlotta AR.<br>Lancet Digit Health. 2023 Jul;5(7):e435-e445. doi: 10.1016/S2589-7500(23)00067-5. Epub 2023 May 19.<br>PMID: 37211455	2	1	1	2	1	1	2
Prediction of Ureteral Injury During Colorectal Surgery Using Machine Learning.<br>Chen KA, Joisa CU, Stem JM, Guillem JG, Gomez SM, Kapadia MR.<br>Am Surg. 2023 Dec;89(12):5702-5710. doi: 10.1177/00031348231173981. Epub 2023 May 3.<br>PMID: 37133432	3	2	1	3	2	1	2

Using artificial intelligence to reduce orthopedic surgical site infection surveillance workload: Algorithm design, validation, and implementation in 4 Spanish hospitals.<br>Flores-Balado Á, Castresana Méndez C, Herrero González A, Mesón Gutierrez R, de Las Casas Cámara G, Vila Cordero B, Arcos J, Pfang B, Martín-Ríos MD; Surgical Site Infection Surveillance Group.<br>Am J Infect Control. 2023 Nov;51(11):1225-1229. doi: 10.1016/j.ajic.2023.04.165. Epub 2023 Apr 24.<br>PMID: 37100291	2	1	1	2	1	1	2
Prediction of Early Visual Outcome of Small-Incision Lenticule Extraction (SMILE) Based on Deep Learning.<br>Wan Q, Yue S, Tang J, Wei R, Tang J, Ma K, Yin H, Deng YP.<br>Ophthalmol Ther. 2023 Apr;12(2):1263-1279. doi: 10.1007/s40123-023-00680-6. Epub 2023 Feb 24.<br>PMID: 36826752	2	1	1	1	2	1	1
Reliability of Postoperative Free Flap Monitoring with a Novel Prediction Model Based on Supervised Machine Learning.<br>Huang RW, Tsai TY, Hsieh YH, Hsu CC, Chen SH, Lee CH, Lin YT, Kao HK, Lin CH.<br>Plast Reconstr Surg. 2023 Nov 1;152(5):943e-952e. doi: 10.1097/PRS.0000000000010307. Epub 2023 Feb 15.<br>PMID: 36790782	2	1	2	1	3	2	1
Prognostic artificial intelligence model to predict 5 year survival at 1 year after gastric cancer surgery based on nutrition and body morphometry.<br>Chung H, Ko Y, Lee IS, Hur H, Huh J, Han SU, Kim KW, Lee J.<br>J Cachexia Sarcopenia Muscle. 2023 Apr;14(2):847-859. doi: 10.1002/jcsm.13176. Epub 2023 Feb 12.<br>PMID: 36775841	2	3	1	1	2	2	1
Recognition of Postoperative Cystography Features by Artificial Intelligence to Predict Recovery from Postprostatectomy Urinary Incontinence: A Rapid and Easy Way to Predict Functional Outcome.<br>Shao IH, Kan HC, Chen HY, Chang YH, Huang LK, Chu YC, Lin PH, Yu KJ, Chuang CK, Pang ST, Wu CT.<br>J Pers Med. 2023 Jan 8;13(1):126. doi: 10.3390/jpm13010126.<br>PMID: 36675787	3	2	1	1	2	1	2

Stratification of Length of Stay Prediction following Surgical Cytoreduction in Advanced High-Grade Serous Ovarian Cancer Patients Using Artificial Intelligence; the Leeds L-AI-OS Score.<br>Laios A, De Freitas DLD, Saalmink G, Tan YS, Johnson R, Zubayraeva A, Munot S, Hutson R, Thangavelu A, Broadhead T, Nugent D, Kalampokis E, de Lima KMG, Theophilou G, De Jong D.<br>Curr Oncol. 2022 Nov 23;29(12):9088-9104. doi: 10.3390/curroncol29120711.<br>PMID: 36547125	2	2	1	1	2	2	2
Prediction Model for 30-Day Mortality after Non-Cardiac Surgery Using Machine-Learning Techniques Based on Preoperative Evaluation of Electronic Medical Records.<br>Choi B, Oh AR, Lee SH, Lee DY, Lee JH, Yang K, Kim HY, Park RW, Park J.<br>J Clin Med. 2022 Nov 1;11(21):6487. doi: 10.3390/jcm11216487.<br>PMID: 36362715	2	2	1	1	2	1	2
Machine-learning model predicting postoperative delirium in older patients using intraoperative frontal electroencephalographic signatures.<br>Röhr V, Blankertz B, Radtke FM, Spies C, Koch S.<br>Front Aging Neurosci. 2022 Oct 14;14:911088. doi: 10.3389/fnagi.2022.911088. eCollection 2022.<br>PMID: 36313029	2	2	1	1	2	1	2
Application of supervised machine learning algorithms to predict the risk of hidden blood loss during the perioperative period in thoracolumbar burst fracture patients complicated with neurological compromise.<br>Yang B, Gao L, Wang X, Wei J, Xia B, Liu X, Zheng P.<br>Front Public Health. 2022 Sep 26;10:969919. doi: 10.3389/fpubh.2022.969919. eCollection 2022.<br>PMID: 36225767	3	3	1	1	3	3	3
Development and Validation of Machine Learning Models to Predict Readmission After Colorectal Surgery.<br>Chen KA, Joisa CU, Stitzenberg KB, Stem J, Guillem JG, Gomez SM, Kapadia MR.<br>J Gastrointest Surg. 2022 Nov;26(11):2342-2350. doi: 10.1007/s11605-022-05443-5. Epub 2022 Sep 7.<br>PMID: 36070116	1	1	1	1	2	1	1

Application of Machine Learning Models to Predict Recurrence After Surgical Resection of Nonmetastatic Renal Cell Carcinoma.<br>Khene ZE, Bigot P, Doumerc N, Ouzaid I, Boissier R, Nouhaud FX, Albiges L, Bernhard JC, Ingels A, Borchiellini D, Kammerer-Jacquet S, Rioux-Leclercq N, Roupret M, Acosta O, De Crevoisier R, Bensalah K; Collaborators.<br>Eur Urol Oncol. 2023 Jun;6(3):323-330. doi: 10.1016/j.euo.2022.07.007. Epub 2022 Aug 18.<br>PMID: 35987730	2	2	1	1	2	1	1
Multi-center validation of machine learning model for preoperative prediction of postoperative mortality.<br>Lee SW, Lee HC, Suh J, Lee KH, Lee H, Seo S, Kim TK, Lee SW, Kim YJ.<br>NPJ Digit Med. 2022 Jul 12;5(1):91. doi: 10.1038/s41746-022-00625-6.<br>PMID: 35821515	2	1	1	1	2	1	1
Development and validation of machine learning models for prediction of seizure outcome after pediatric epilepsy surgery.<br>Yossofzai O, Fallah A, Maniquis C, Wang S, Ragheb J, Weil AG, Brunette-Clement T, Andrade A, Ibrahim GM, Mitsakakis N, Widjaja E.<br>Epilepsia. 2022 Aug;63(8):1956-1969. doi: 10.1111/epi.17320. Epub 2022 Jun 25.<br>PMID: 35661152	2	2	1	1	2	1	1
Robust Prediction of Non-home Discharge After Thoracolumbar Spine Surgery With Ensemble Machine Learning and Validation on a Nationwide Cohort.<br>Valliani AA, Kim NC, Martini ML, Gal JS, Neifert SN, Feng R, Geng EA, Kim JS, Cho SK, Oermann EK, Caridi JM.<br>World Neurosurg. 2022 Sep;165:e83-e91. doi: 10.1016/j.wneu.2022.05.105. Epub 2022 May 30.<br>PMID: 35654334	2	2	1	1	3	1	1
Using Artificial Intelligence to Find the Optimal Margin Width in Hepatectomy for Colorectal Cancer Liver Metastases.<br>Bertsimas D, Margonis GA, Sujichantararat S, Boerner T, Ma Y, Wang J, Kamphues C, Sasaki K, Tang S, Gagniere J, Dupré A, Løes IM, Wagner D, Stasinos G, Macher-Beer A, Burkhart R, Morioka D, Imai K, Ardiles V, O'Connor JM, Pawlik TM, Poultsides G, Seeliger H, Beyer K, Kaczirek K, Kornprat P, Aucejo FN, de Santibañes E, Baba H, Endo I, Lønning PE, Kreis ME, Weiss MJ, Wolfgang CL, D'Angelica M.<br>JAMA Surg. 2022 Aug 1;157(8):e221819.	3	2	1	1	2	1	1

doi: 10.1001/jamasurg.2022.1819. Epub 2022 Aug 10.<br>PMID: 35648428							
Development and Validation of an Explainable Machine Learning Model for Major Complications After Cytoreductive Surgery.<br>Deng H, Eftekhari Z, Carlin C, Veerapong J, Fournier KF, Johnston FM, Dineen SP, Powers BD, Hendrix R, Lambert LA, Abbott DE, Vande Walle K, Grotz TE, Patel SH, Clarke CN, Staley CA, Abdel-Misih S, Cloyd JM, Lee B, Fong Y, Raoof M.<br>JAMA Netw Open. 2022 May 2;5(5):e2212930. doi: 10.1001/jamanetworkopen.2022.12930.<br>PMID: 35612856	2	2	1	1	2	1	1
Development and validation of an automated planning tool for navigated lumbosacral pedicle screws using a convolutional neural network.<br>Scherer M, Kausch L, Ishak B, Norajitra T, Vollmuth P, Kiening K, Unterberg A, Maier-Hein K, Neumann JO.<br>Spine J. 2022 Oct;22(10):1666-1676. doi: 10.1016/j.spinee.2022.05.002. Epub 2022 May 16.<br>PMID: 35584757	2	2	1	1	1	1	1
Optimizing discharge after major surgery using an artificial intelligence-based decision support tool (DESIRE): An external validation study.<br>van de Sande D, van Genderen ME, Verhoef C, Huiskens J, Gommers D, van Unen E, Schasfoort RA, Schepers J, van Bommel J, Grünhagen DJ.<br>Surgery. 2022 Aug;172(2):663-669. doi: 10.1016/j.surg.2022.03.031. Epub 2022 May 4.<br>PMID: 35525621	2	2	1	1	2	1	1

Novel "resect and analysis" approach for T2 colorectal cancer with use of artificial intelligence.<br>Ichimasa K, Nakahara K, Kudo SE, Misawa M, Bretthauer M, Shimada S, Takehara Y, Mukai S, Kouyama Y, Miyachi H, Sawada N, Mori K, Ishida F, Mori Y.<br>Gastrointest Endosc. 2022 Oct;96(4):665-672.e1. doi: 10.1016/j.gie.2022.04.1305. Epub 2022 Apr 30.<br>PMID: 35500659	3	2	1	1	2	1	1
Can We Geographically Validate a Natural Language Processing Algorithm for Automated Detection of Incidental Durotomy Across Three Independent Cohorts From Two Continents?<br>Karhade AV, Oosterhoff JHF, Groot OQ, Agaronnik N, Ehresman J, Bongers MER, Jaarsma RL, Poonnoose SI, Sciubba DM, Tobert DG, Doornberg JN, Schwab JH.<br>Clin Orthop Relat Res. 2022 Sep 1;480(9):1766-1775. doi: 10.1097/CORR.0000000000002200. Epub 2022 Apr 12.<br>PMID: 35412473	2	3	1	1	1	1	1
Prediction of complications and surgery duration in primary TKA with high accuracy using machine learning with arthroplasty-specific data.<br>Hinterwimmer F, Lazic I, Langer S, Suren C, Charitou F, Hirschmann MT, Matziolis G, Seidl F, Pohlig F, Rueckert D, Burgkart R, von Eisenhart-Rothe R.<br>Knee Surg Sports Traumatol Arthrosc. 2023 Apr;31(4):1323-1333. doi: 10.1007/s00167-022-06957-w. Epub 2022 Apr 8.<br>PMID: 35394135	3	2	1	1	2	1	1
Development and internal validation of machine learning algorithms to predict patient satisfaction after total hip arthroplasty.<br>Zhang S, Chen JY, Pang HN, Lo NN, Yeo SJ, Liow MHL.<br>Arthroplasty. 2021 Sep 2;3(1):33. doi: 10.1186/s42836-021-00087-3.<br>PMID: 35236492	3	2	1	1	2	2	1
Artificial Intelligence Based Machine Learning Models Predict Sperm Parameter Upgrading after Varicocele Repair: A Multi-Institutional Analysis.<br>Ory J, Tradewell MB, Blankstein U, Lima TF, Nackeeran S, Gonzalez DC, Nwefo E, Moryousef J, Madhusoodanan V, Lau S, Jarvi K, Ramasamy R.<br>World J Mens Health. 2022 Oct;40(4):618-626. doi: 10.5534/wjmh.210159. Epub	2	2	1	1	2	1	1

2022 Jan 2.<br>PMID: 35021305							
Machine learning algorithm to predict anterior cruciate ligament revision demonstrates external validity.<br>Martin RK, Wastvedt S, Pareek A, Persson A, Visnes H, Fenstad AM, Moatshe G, Wolfson J, Lind M, Engebretsen L.<br>Knee Surg Sports Traumatol Arthrosc. 2022 Feb;30(2):368-375. doi: 10.1007/s00167-021-06828-w. Epub 2022 Jan 1.<br>PMID: 34973096	2	2	1	1	2	1	1
Artificial Intelligence Supports Decision Making during Open-Chest Surgery of Rare Congenital Heart Defects.<br>Lo Muzio FP, Rozzi G, Rossi S, Luciani GB, Foresti R, Cabassi A, Fassina L, Miragoli M.<br>J Clin Med. 2021 Nov 16;10(22):5330. doi: 10.3390/jcm10225330.<br>PMID: 34830612	3	2	1	1	1	1	1
A Novel Predictive Model for Anastomotic Leakage in Colorectal Cancer Using Auto-artificial Intelligence.<br>Mazaki J, Katsumata K, Ohno Y, Udo R, Tago T, Kasahara K, Kuwabara H, Enomoto M, Ishizaki T, Nagakawa Y, Tsuchida A.<br>Anticancer Res. 2021 Nov;41(11):5821-5825. doi: 10.21873/anticanres.15400.<br>PMID: 34732457	2	2	1	1	2	1	1
Application of Machine Learning Algorithms to Predict Clinically Meaningful Improvement After Arthroscopic Anterior Cruciate Ligament Reconstruction.<br>Kunze KN, Polce EM, Ranawat AS, Randsborg PH, Williams RJ 3rd, Allen AA, Nwachukwu BU; HSS ACL Registry Group; Pearle A, Stein BS, Dines D, Kelly A, Kelly B, Rose H, Maynard M, Strickland S, Coleman S, Hannafin J, MacGillivray J, Marx R, Warren R, Rodeo S, Fealy S, O'Brien S, Wickiewicz T, Dines JS, Cordasco F, Altcheck D.<br>Orthop J Sports Med. 2021 Oct 14;9(10):23259671211046575. doi: 10.1177/23259671211046575. eCollection 2021 Oct.<br>PMID: 34671691	2	2	1	1	2	1	1

Artificial Intelligence May Predict Early Sepsis After Liver Transplantation.<br>Kamaleswaran R, Sataphaty SK, Mas VR, Eason JD, Maluf DG.<br>Front Physiol. 2021 Sep 6;12:692667. doi: 10.3389/fphys.2021.692667. eCollection 2021.<br>PMID: 34552499	2	2	1	1	2	1	1
Machine Learning to Predict Fascial Dehiscence after Exploratory Laparotomy Surgery.<br>Cole J, Hughey S, Metzger A, Geiger P, Fluke L, Booth GJ.<br>J Surg Res. 2021 Dec;268:514-520. doi: 10.1016/j.jss.2021.06.068. Epub 2021 Aug 26.<br>PMID: 34455314	2	1	1	1	1	1	1
Using a Convolutional Neural Network to Predict Remission of Diabetes After Gastric Bypass Surgery: Machine Learning Study From the Scandinavian Obesity Surgery Register.<br>Cao Y, Näslund I, Näslund E, Ottosson J, Montgomery S, Stenberg E.<br>JMIR Med Inform. 2021 Aug 19;9(8):e25612. doi: 10.2196/25612.<br>PMID: 34420921	2	1	1	1	1	1	1
Machine Learning to Improve Prognosis Prediction of Early Hepatocellular Carcinoma After Surgical Resection.<br>Ji GW, Fan Y, Sun DW, Wu MY, Wang K, Li XC, Wang XH.<br>J Hepatocell Carcinoma. 2021 Aug 10;8:913-923. doi: 10.2147/JHC.S320172. eCollection 2021.<br>PMID: 34414136	2	1	1	1	2	1	1
Development of novel artificial intelligence systems to predict facial morphology after orthognathic surgery and orthodontic treatment in Japanese patients.<br>Tanikawa C, Yamashiro T.<br>Sci Rep. 2021 Aug 4;11(1):15853. doi: 10.1038/s41598-021-95002-w.<br>PMID: 34349151	3	2	1	1	2	2	1
Machine Learning Algorithm Using Electronic Chart-Derived Data to Predict Delirium After Elderly Hip Fracture Surgeries: A Retrospective Case-Control Study.<br>Zhao H, You J, Peng Y, Feng Y.<br>Front Surg. 2021 Jul 13;8:634629. doi: 10.3389/fsurg.2021.634629. eCollection 2021.<br>PMID: 34327210	3	2	1	1	1	2	1

Establishing Machine Learning Models to Predict Curative Resection in Early Gastric Cancer with Undifferentiated Histology: Development and Usability Study.<br>Bang CS, Ahn JY, Kim JH, Kim YI, Choi IJ, Shin WG.<br>J Med Internet Res. 2021 Apr 15;23(4):e25053. doi: 10.2196/25053.<br>PMID: 33856358	3	2	1	1	2	1	1
Early identification of epilepsy surgery candidates: A multicenter, machine learning study.<br>Wissel BD, Greiner HM, Glauser TA, Pestian JP, Kemme AJ, Santel D, Ficker DM, Mangano FT, Szczesniak RD, Dexheimer JW.<br>Acta Neurol Scand. 2021 Jul;144(1):41-50. doi: 10.1111/ane.13418. Epub 2021 Mar 26.<br>PMID: 33769560	3	2	1	1	2	1	1
Validation of the Artificial Intelligence-Based Predictive Optimal Trees in Emergency Surgery Risk (POTTER) Calculator in Emergency General Surgery and Emergency Laparotomy Patients.<br>El Hechi MW, Maurer LR, Levine J, Zhuo D, El Moheb M, Velmahos GC, Dunn J, Bertsimas D, Kaafarani HM.<br>J Am Coll Surg. 2021 Jun;232(6):912-919.e1. doi: 10.1016/j.jamcollsurg.2021.02.009. Epub 2021 Mar 8.<br>PMID: 33705983	3	2	1	1	2	1	1
Validation of the AI-based Predictive OpTimal Trees in Emergency Surgery Risk (POTTER) Calculator in Patients 65 Years and Older.<br>Maurer LR, Chetlur P, Zhuo D, El Hechi M, Velmahos GC, Dunn J, Bertsimas D, Kaafarani HMA.<br>Ann Surg. 2023 Jan 1;277(1):e8-e15. doi: 10.1097/SLA.0000000000004714. Epub 2020 Dec 23.<br>PMID: 33378309	2	2	1	1	2	1	1
Artificial intelligence facilitates decision-making in the treatment of lumbar disc herniations.<br>Wirries A, Geiger F, Hammad A, Oberkircher L, Blümcke I, Jabari S.<br>Eur Spine J. 2021 Aug;30(8):2176-2184. doi: 10.1007/s00586-020-06613-2. Epub 2020 Oct 13.<br>PMID: 33048249	2	2	1	1	1	1	1

Machine Learning Algorithms for Predicting and Risk Profiling of Cardiac Surgery-Associated Acute Kidney Injury.<br>Penny-Dimri JC, Bergmeir C, Reid CM, Williams-Spence J, Cochrane AD, Smith JA.<br>Semin Thorac Cardiovasc Surg. 2021 Autumn;33(3):735-745. doi: 10.1053/j.semtcvs.2020.09.028. Epub 2020 Sep 24.<br>PMID: 32979479	2	2	1	1	2	1	1
Explainable Machine Learning Approach as a Tool to Understand Factors Used to Select the Refractive Surgery Technique on the Expert Level.<br>Yoo TK, Ryu IH, Choi H, Kim JK, Lee IS, Kim JS, Lee G, Rim TH.<br>Transl Vis Sci Technol. 2020 Feb 12;9(2):8. doi: 10.1167/tvst.9.2.8.<br>PMID: 32704414	3	2	1	1	2	1	1
Accuracy of a new intraocular lens power calculation method based on artificial intelligence.<br>Carmona González D, Palomino Bautista C.<br>Eye (Lond). 2021 Feb;35(2):517-522. doi: 10.1038/s41433-020-0883-3. Epub 2020 Apr 28.<br>PMID: 32346109	3	2	1	1	2	1	1
Development and Validation of Machine Learning Algorithms for Predicting Adverse Events After Surgery for Lumbar Degenerative Spondylolisthesis.<br>Fatima N, Zheng H, Massaad E, Hadzipasic M, Shankar GM, Shin JH.<br>World Neurosurg. 2020 Aug;140:627-641. doi: 10.1016/j.wneu.2020.04.135. Epub 2020 Apr 25.<br>PMID: 32344139	3	2	1	1	2	1	1
Development of machine learning and natural language processing algorithms for preoperative prediction and automated identification of intraoperative vascular injury in anterior lumbar spine surgery.<br>Karhade AV, Bongers MER, Groot OQ, Cha TD, Doorly TP, Fogel HA, Hershman SH, Tobert DG, Srivastava SD, Bono CM, Kang JD, Harris MB, Schwab JH.<br>Spine J. 2021 Oct;21(10):1635-1642. doi: 10.1016/j.spinee.2020.04.001. Epub 2020 Apr 12.<br>PMID: 32294557	3	2	1	1	2	1	1

Can natural language processing provide accurate, automated reporting of wound infection requiring reoperation after lumbar discectomy?<br>Karhade AV, Bongers MER, Groot OQ, Cha TD, Doorly TP, Fogel HA, Hershman SH, Tobert DG, Schoenfeld AJ, Kang JD, Harris MB, Bono CM, Schwab JH.<br>Spine J. 2020 Oct;20(10):1602-1609. doi: 10.1016/j.spinee.2020.02.021. Epub 2020 Mar 4.<br>PMID: 32145358	3	2	1	1	2	1	1
Using artificial intelligence (AI) to predict postoperative surgical site infection: A retrospective cohort of 4046 posterior spinal fusions.<br>Hopkins BS, Mazmudar A, Driscoll C, Svet M, Goergen J, Kelsten M, Shlobin NA, Kesavabhotla K, Smith ZA, Dahdaleh NS.<br>Clin Neurol Neurosurg. 2020 May;192:105718. doi: 10.1016/j.clineuro.2020.105718. Epub 2020 Feb 3.<br>PMID: 32065943	2	1	1	1	2	2	1
Natural language processing for automated detection of incidental durotomy.<br>Karhade AV, Bongers MER, Groot OQ, Kazarian ER, Cha TD, Fogel HA, Hershman SH, Tobert DG, Schoenfeld AJ, Bono CM, Kang JD, Harris MB, Schwab JH.<br>Spine J. 2020 May;20(5):695-700. doi: 10.1016/j.spinee.2019.12.006. Epub 2019 Dec 23.<br>PMID: 31877390	2	1	1	1	2	2	1
Using machine learning to predict 30-day readmissions after posterior lumbar fusion: an NSQIP study involving 23,264 patients.<br>Hopkins BS, Yamaguchi JT, Garcia R, Kesavabhotla K, Weiss H, Hsu WK, Smith ZA, Dahdaleh NS.<br>J Neurosurg Spine. 2019 Nov 29;32(3):399-406. doi: 10.3171/2019.9.SPINE19860. Print 2020 Mar 1.<br>PMID: 31783353	3	2	1	1	2	1	1
Predicting nonroutine discharge after elective spine surgery: external validation of machine learning algorithms.<br>Stopa BM, Robertson FC, Karhade AV, Chua M, Broekman MLD, Schwab JH, Smith TR, Gormley WB.<br>J Neurosurg Spine. 2019 Jul 26;31(5):742-747. doi: 10.3171/2019.5.SPINE1987. Print 2019 Nov 1.<br>PMID: 31349223	3	2	1	1	2	1	1

Transfusion after total knee arthroplasty can be predicted using the machine learning algorithm.<br>Jo C, Ko S, Shin WC, Han HS, Lee MC, Ko T, Ro DH.<br>Knee Surg Sports Traumatol Arthrosc. 2020 Jun;28(6):1757-1764. doi: 10.1007/s00167-019-05602-3. Epub 2019 Jun 28.<br>PMID: 31254027	3	2	1	1	2	1	1
Convolutional neural network to predict the local recurrence of giant cell tumor of bone after curettage based on pre-surgery magnetic resonance images.<br>He Y, Guo J, Ding X, van Ooijen PMA, Zhang Y, Chen A, Oudkerk M, Xie X.<br>Eur Radiol. 2019 Oct;29(10):5441-5451. doi: 10.1007/s00330-019-06082-2. Epub 2019 Mar 11.<br>PMID: 30859281	3	3	1	1	2	1	1
Artificial intelligence may help in predicting the need for additional surgery after endoscopic resection of T1 colorectal cancer.<br>Ichimasa K, Kudo SE, Mori Y, Misawa M, Matsudaira S, Kouyama Y, Baba T, Hidaka E, Wakamura K, Hayashi T, Kudo T, Ishigaki T, Yagawa Y, Nakamura H, Takeda K, Haji A, Hamatani S, Mori K, Ishida F, Miyachi H.<br>Endoscopy. 2018 Mar;50(3):230-240. doi: 10.1055/s-0043-122385. Epub 2017 Dec 22.<br>PMID: 29272905	3	3	1	1	2	1	1
Validation of an online risk calculator for the prediction of anastomotic leak after colon cancer surgery and preliminary exploration of artificial intelligence-based analytics.<br>Sammour T, Cohen L, Karunatillake AI, Lewis M, Lawrence MJ, Hunter A, Moore JW, Thomas ML.<br>Tech Coloproctol. 2017 Nov;21(11):869-877. doi: 10.1007/s10151-017-1701-1. Epub 2017 Oct 28.<br>PMID: 29080956	3	2	1	1	2	1	1
Artificial intelligence versus logistic regression statistical modelling to predict cardiac complications after noncardiac surgery.<br>Lette J, Colletti BW, Cerino M, McNamara D, Eybalin MC, Levasseur A, Nattel S.<br>Clin Cardiol. 1994 Nov;17(11):609-14. doi: 10.1002/clc.4960171109.<br>PMID: 7834935	2	1	1	1	2	1	1

Table S1: ROBINS-I.
